# Small Bowel Obstruction Following Dislodgement of an Intragastric Balloon: A Case Report

**DOI:** 10.7759/cureus.67738

**Published:** 2024-08-25

**Authors:** Laura Brizuela, Hani Samarah, Nicole Cardona

**Affiliations:** 1 Internal Medicine, Florida International University, Herbert Wertheim College of Medicine, Miami, USA; 2 Medicine, Herbert Wertheim College of Medicine, Miami, USA; 3 Internal Medicine, Baptist Hospital of Miami, Miami, USA

**Keywords:** deflated intragastric balloon, small bowel obstruction, orbera balloon, obesity interventional treatments, intragastric balloon removal

## Abstract

Obesity remains a significant health burden worldwide, requiring diverse and effective treatment strategies. The intragastric balloon (IGB), developed in the 1980s, offers a non-surgical option for weight management. Despite a decrease in usage, the IGB procedure continues to be an option for patients both domestically and abroad. In this article, we present the case involving a 30-year-old female who presented with severe abdominal complications 18 months after IGB placement in the Dominican Republic, well beyond the recommended six-month period for removal. This case highlights the critical risks associated with delayed IGB removal, including balloon rupture, migration, and symptoms indicative of gastric outlet obstruction. The literature supports increased complication rates with prolonged balloon retention, including risks such as gastric perforation, ulceration, and small bowel obstruction, emphasizing the importance of adhering to removal schedules. Furthermore, the case stresses the need for psychosocial evaluations before weight loss procedures and the necessity of alternative methods like laparoscopic removal when endoscopic extraction is unsuccessful. As obesity management evolves with new treatments like glucagon-like peptide-1 (GLP-1) analogs, ongoing research to understand their interaction with IGBs is crucial. This case underlines the importance of rigorous follow-up care, educating patients about procedural timelines, and conducting comprehensive evaluations to ensure the safety and effectiveness of IGB therapy.

## Introduction

Obesity, classified by body mass index (BMI) criteria set by the World Health Organization (WHO), is a significant global public health concern. Its prevalence has been rising alarmingly, with the WHO reporting more than 1.9 billion adults, 18 years and older, were overweight, of which over 650 million were obese. This indicates that approximately 39% of adults aged 18 and above were overweight, and 13% were obese. Notably, obesity rates are higher among women, with 15% of women being obese compared to 11% of men. The trend extends beyond adults, affecting children and adolescents, underscoring the urgency for effective interventions. The WHO's statistics highlight the severity of the obesity epidemic, emphasizing its role as a major contributor to global morbidity and mortality due to its association with numerous chronic diseases, including diabetes, cardiovascular diseases, and certain cancers [1].

A myriad of weight loss methods has been developed to combat the global obesity epidemic, ranging from non-surgical approaches, such as lifestyle modifications, dietary changes, and pharmacotherapy, to surgical interventions, such as bariatric surgery [1]. Non-surgical methods, often the first line of treatment, focus on diet, exercise, and behavioral therapy but may also include the use of anti-obesity medications. Despite their initial appeal due to minimal invasiveness, their efficacy can be limited over time, driving individuals toward more definitive surgical options. For instance, bariatric surgery has gained popularity for its long-term effectiveness, with studies showing weight loss of 20%-35% maintained for over 10 years, and the substantial reduction of obesity-related comorbidities [2]. However, among the array of interventional treatment options, the intragastric balloon (IGB) procedure stands out as a minimally invasive, temporary method for weight reduction. Introduced in the early 1980s, the IGB procedure has evolved, offering a bridge between non-surgical weight loss methods and surgery for individuals struggling with obesity [3]. This procedure involves the endoscopic placement of a balloon in the stomach, which is filled with saline to induce satiety, thereby limiting food intake and promoting weight loss over several months [3]. The popularity of the IGB procedure stems from its non-surgical nature, reversibility, and role in facilitating lifestyle changes toward sustainable weight management [4]. However, despite its benefits, the IGB procedure is not without risks; potential complications such as gastric discomfort, ulceration, and, in rare cases, bowel obstruction highlight the need for careful patient selection and monitoring [4].

The efficacy of the IGB procedure in facilitating significant weight loss has been documented, with patients typically experiencing a reduction of 10%-15% in body weight within the first six months post-implantation [3]. In a study examining obesity management in 652,927 patients, a total of 2,910 IGB procedures were performed, indicating the utilization of the IGB procedure among the available options for weight management. However, the study also highlighted a significant decline in the number of IGB procedures between 2016 and 2019, from 953 procedures in 2016 to 418 procedures in 2019, suggesting a shift in the preference or availability of obesity treatment methods during this period. Currently, IGB procedures represent a much smaller proportion, typically around 1%-3% of all bariatric interventions, compared to other interventions such as gastric sleeve and bypass [5]. Although the procedure is associated with a low rate of serious adverse events, vigilant patient monitoring is crucial to manage potential complications, enhancing the procedure's safety and efficacy [3].

Despite the success, the procedure is not without risks; an analysis conducted by Tate and Geliebter found the occurrence of adverse events following the placement of intragastric balloons to be 28.2%. These adverse events include nausea, vomiting, and abdominal pain, while more serious adverse events, documented at 10.5%, include gastric perforation, severe pancreatitis, and small bowel obstruction [6]. Specific to small bowel obstruction (SBO), this serious complication is exceedingly rare, with reported incidences of less than 0.1% [7]. Further, there are multiple IGB systems on the market, each with its own safety profiles. Similar to the case presented here, the Orbera Intragastric Balloon (IGB) System was systematically evaluated by the American Society for Gastrointestinal Endoscopy (ASGE) Bariatric Endoscopy Task Force, showcasing a safety profile with serious adverse events occurring in less than 5% of cases [7]. The management of complications is tailored to their severity, from conservative approaches for mild symptoms to the necessity of endoscopic or surgical interventions in cases of significant adverse events such as SBO. This underscores the importance of vigilant patient monitoring and the prompt addressing of any complications to mitigate risks and enhance the procedure's safety and efficacy.

## Case presentation

The patient is a 30-year-old Hispanic female with no significant past medical history, who had an intragastric Orbera balloon placed for weight loss 18 months ago in the Dominican Republic. She presented to the emergency department with worsening abdominal pain for two days and a new onset of vomiting for the past 24 hours. She described the pain as progressively worsening, diffuse, mainly in the epigastric area, and radiating to the right upper quadrant. The pain started two days prior, and she rated it as 10 out of 10. She also mentioned that she had been experiencing similar abdominal pain over the past year, but the pain was severe this time; she initially thought it was related to her gallbladder. Emesis started 24 hours prior, with a total of 17 episodes. She described her emesis as bilious and non-bloody. She denied any recent travels or dietary changes and did not think the abdominal pain and emesis were related to foods. She also stated not having bowel movements for the last two days but noted passing flatus. Additionally, she denied any signs of gastrointestinal bleeding, urinary changes, fever, chills, and diarrhea. The patient also reported cannabis use.

On arrival, the patient appeared in mild distress due to abdominal discomfort, but her vital signs were stable. Abdominal examination demonstrated normal bowel sounds, diffuse abdominal tenderness without rebound tenderness, guarding or rigidity, and soft, mild left-sided distension. McBurney, Rovsing, Psoas, and Murphy's signs were negative. Labs on admission showed white blood cells at 14.16 × 10^3^/mcL, hemoglobin at 13.3 g/dL, hematocrit at 41.1%, platelets at 350,000/mcL, INR at 1.1, and BMI at 36.21 kg/m².

**Figure 1:**
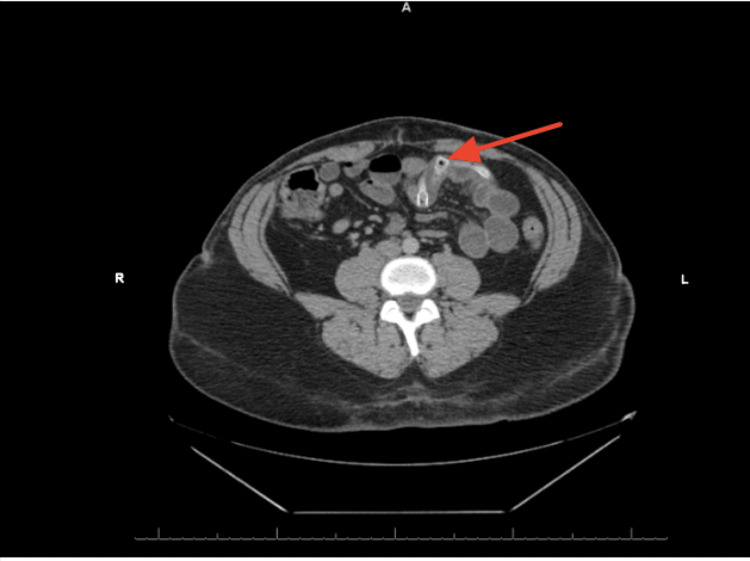
The axial view of a metallic deflated structure in the small bowel at the mid-abdomen showing mildly dilated bowel loops

**Figure 2:**
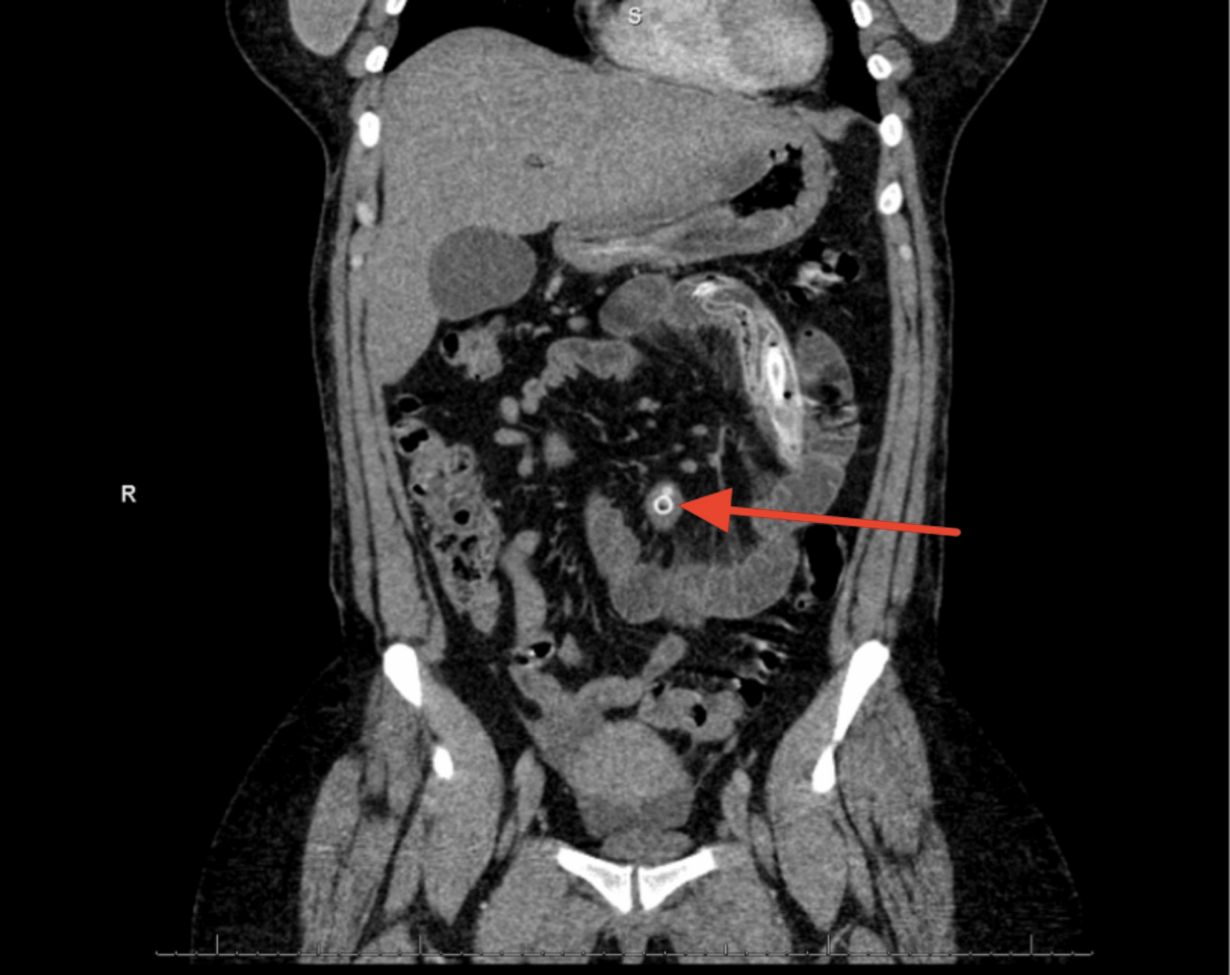
The coronal view of a deflated structure in the small bowel at the mid-abdomen showing the mildly dilated bowel loops

On admission, computed tomography of the abdomen and pelvis with intravenous contrast demonstrated a metallic deflated structure in the small bowel at the mid-abdomen. Mildly dilated bowel loops were observed, associated with partial SBO. The findings were consistent with an SBO, following the spontaneous deflation and migration of an IGB. Furthermore, the studies showed no signs of free fluid or air in the abdomen (Figures 1, 2).

Throughout the patient's hospital stay, the gastroenterology team was consulted and planned a single balloon enteroscopy, intending to potentially remove the foreign body from the small intestine. The surgery team was also on standby to provide support if endoscopic treatment was unsuccessful and surgical intervention became necessary. The patient was informed of all available treatment options, including both surgical and non-surgical management approaches. After discussing these options, the patient chose to proceed with endoscopic removal as the initial approach.

However, the gastroenterology team was unable to retrieve the foreign object from the small intestine. Enteroscopy was not performed on the mid-jejunum as it appeared normal endoscopically. Subsequently, the surgery team performed an exploratory laparotomy by opening the small bowel, successfully retrieving the foreign body. The patient tolerated the procedure well and was discharged home five days later.

## Discussion

This case report describes a 30-year-old female patient who experienced severe abdominal pain and vomiting 18 months post Orbera IGB placement, a device typically recommended for removal six months after insertion. The prolonged retention beyond the advised period underscores a significant deviation from standard clinical guidelines, which suggest a maximum in-situ duration of six months to minimize risks [8]. Recent studies underscore the heightened risk associated with the prolonged retention of IGBs beyond the recommended six-month period. For instance, Fittipaldi-Fernandez et al. observed that complications such as balloon rupture and migration could increase significantly with extended IGB placement [9]. Specifically, the incidence of adverse events related to IGBs retained for over six months was reported as high as 32% compared to a much lower rate of 2.3% for those adhering to the recommended time frame. Among these complications, gastric obstruction and spontaneous balloon hyperinflation were particularly noteworthy, presenting serious health risks that necessitated urgent medical intervention [10,11]. The case presented here adds to the literature and illustrates the potential increase in complication rates with delayed IGB removal [11,12].

IGB devices are known for their role in weight management strategies, providing a non-surgical option for individuals struggling with obesity. While the literature extensively documents the short-term safety and efficacy of these devices [12,13], instances of prolonged retention and associated complications are less frequently highlighted. Studies have reported an increase in adverse events, such as balloon rupture and migration, with balloons retained beyond six months [9,10,14]. This case aligns with those findings, demonstrating the heightened risks of serious complications from extended IGB placement.

Taking into consideration the risks associated with the deviation from recommended treatment plans, this case also indicates that psychosocial evaluation should be considered before recommending weight loss interventions. This case underscores the necessity of strict adherence to the recommended IGB removal timeline and the importance of comprehensive patient education and follow-up care. It also highlights the need for healthcare providers to discuss the potential psychological motivations and expectations of patients seeking weight loss interventions, particularly when obtained abroad, as seen in this case. This comprehensive approach can help mitigate risks and ensure timely intervention in the event of complications.

Laparoscopic removal of IGBs emerges as a viable alternative when endoscopic extraction fails, typically due to technical challenges or complications such as severe fibrosis around the balloon or balloon deflation [13], as presented in this case. This method should be considered when the balloon is excessively inflated, deformed, or adhered to the gastric wall, conditions that significantly increase the risk of obstruction and perforation [13]. Laparoscopic intervention allows for the safe and effective removal of the balloon under direct visualization, minimizing the risk of gastric damage. The procedure is particularly recommended in cases where endoscopic attempts have been unsuccessful or when the patient presents with acute symptoms indicative of significant gastric distress or obstruction [13].

Further research is vital to explore the long-term safety profile of IGBs, especially considering the evolving landscape of obesity management, which now includes pharmacological interventions like glucagon-like peptide-1 (GLP-1) analogs. The concomitant use of GLP-1 analogs and IGB placement represents an area of clinical interest, given the potential for exacerbated complication rates due to altered gastrointestinal motility and satiety signals. Understanding the interactions between these treatments is crucial for developing comprehensive guidelines that can safely accommodate the integration of pharmacological and device-based interventions.

Given the propensity for patients to deviate from recommended treatment protocols, as demonstrated in this case, there is a clear need for enhanced vigilance in patient follow-up. This underscores the importance of conducting thorough psychological and social evaluations before recommending and pursuing weight loss strategies. Such assessments can help identify individuals who may be at increased risk of non-compliance with post-procedure guidelines, thereby enabling the implementation of targeted follow-up and support mechanisms. By prioritizing research in these areas, the medical community can improve patient selection processes, tailor follow-up care, and minimize the risk of severe complications associated with IGB therapy and concomitant treatments.

## Conclusions

The case presented here enduring severe complications 18 months post Orbera IGB insertion magnifies the critical importance of adhering to the six-month removal guideline to avert substantial risks. This instance not only reinforces the documented necessity for diligent patient follow-up and education regarding the timeliness of IGB removal but also highlights the less discussed, yet significant, psychosocial aspects influencing patient adherence to post-procedural care. Moreover, the successful laparoscopic retrieval after the failure of endoscopic methods underlines the need for alternative approaches in complicated cases, ensuring patient safety and efficacy of the treatment. As the landscape of obesity management evolves with the integration of pharmacological options like GLP-1 analogs, this case underscores the imperative for ongoing research to understand the interactions between different treatment modalities better. This knowledge will be crucial in refining patient selection, enhancing follow-up protocols, and ultimately mitigating the risks associated with IGB therapy. The medical community must remain vigilant, not only in the technical execution of such interventions but also in the holistic assessment of patients, to ensure the highest standards of care and patient safety.
